# Thrombolysis in spontaneous coronary artery dissection: a case report of acute catastrophic dissection requiring coronary artery bypass surgery and delayed heart transplant in a young woman with fibromuscular dysplasia

**DOI:** 10.1093/ehjcr/ytaf517

**Published:** 2025-11-25

**Authors:** Bishow Paudel, Andrew Lenneman, Jose Tallaj, Joanna Joly, Erik J Orozco-Hernandez

**Affiliations:** Department of Medicine, Division of Cardiovascular Medicine, University of Virginia, 1215 Lee Street, Charlottesville, VA 22908, USA; Division of Cardiovascular Medicine, Heart Failure and Transplantation Cardiology, University of Alabama at Birmingham, 1900 University Boulevard, Birmingham, AL 35294, USA; Division of Cardiovascular Medicine, Heart Failure and Transplantation Cardiology, University of Alabama at Birmingham, 1900 University Boulevard, Birmingham, AL 35294, USA; Division of Cardiovascular Medicine, Heart Failure and Transplantation Cardiology, University of Alabama at Birmingham, 1900 University Boulevard, Birmingham, AL 35294, USA; Division of Cardiovascular Medicine, Heart Failure and Transplantation Cardiology, University of Alabama at Birmingham, 1900 University Boulevard, Birmingham, AL 35294, USA

**Keywords:** Case report, Spontaneous Coronary artery dissection (SCAD), Fibromuscular dysplasia (FMD), Superior mesenteric artery (SMA) dissection, Revascularization in SCAD, Post-SCAD heart transplant

## Abstract

**Background:**

Spontaneous coronary artery dissection (SCAD) is a rare condition characterized by a tear in the coronary artery wall and is often associated with fibromuscular dysplasia (FMD). It is unclear whether SCAD with FMD represents a progressive pathology that leads to both acute and chronic complications, including myocardial damage and advanced heart failure.

**Case Summary:**

A 43-year-old woman with chest pain was initially diagnosed with ST-elevation myocardial infarction and later found to have SCAD involving the left main, left anterior descending, and left circumflex arteries. She developed cardiogenic shock requiring emergent coronary artery bypass surgery (CABG). She had a prolonged course with underlying FMD, superior mesenteric artery dissection, multiple decompensated heart failure, and cardiogenic shock ultimately requiring a heart transplant after 2 years.

**Discussion:**

Revascularization in SCAD is not well defined with limited data. The incidence of cardiogenic shock is 1.2%–15.9%. There are case reports on mechanical circulatory support (MCS), CABG, and heart transplantation. This case is unique requiring MCS and CABG with SCAD and heart transplant after 2 years.

Learning points

- Thrombolysis should be avoided in young women with suspected spontaneous coronary artery dissection (SCAD), as it may worsen the dissection.
- Timely recognition of SCAD in low cardiovascular risk patients presenting with ST-elevation myocardial infarction (STEMI) is crucial to prevent complications related to treatment and to ensure optimal management.
- Personalized follow-up is vital for SCAD patients, particularly for those with fibromuscular dysplasia (FMD), genetic variants, or a family history of vascular disease.

## Introduction

Spontaneous coronary artery dissection (SCAD) is a significant and often under-recognized cause of acute coronary syndrome (ACS), particularly in young to middle-aged women who do not exhibit traditional cardiovascular risk factors. While urgent reperfusion is essential in cases of ST-elevation myocardial infarction (STEMI), thrombolytic therapy can actually worsen outcomes in SCAD by exacerbating the dissection or promoting the expansion of intramural haematomas. Despite this risk, current guidelines lack clear recommendations for managing STEMI in patients suspected of having SCAD, who need different management pathways and follow-up.

## Summary figure

**Table:** 

Time	Events
8 July 2021	A 41-year-old woman presented to the emergency department with chest pain and was diagnosed with an anterolateral STEMI. She received systemic tPA and heparin, followed by transfer to a PCI centre.
9 July 2021	Left heart catheterization revealed SCAD involving the LM, LAD, and LCX arteries. The patient developed cardiogenic shock and hypoxic respiratory failure, necessitating mechanical ventilation and placement of an Impella CP device. She subsequently underwent emergent three-vessel CABG.
19 November 2021	PET stress imaging demonstrated no reversible perfusion defects. The LVEF was 40% at rest and 44% during stress. A large fixed perfusion defect was noted in the anteroseptal segment extending from base to apex, with no evidence of inducible ischaemia.
9 December 2021	TTE demonstrated LVEF of 25% with akinesis of the mid to apical septum, Grade III diastolic dysfunction, a moderately dilated left atrium, and moderately reduced right ventricular systolic function. Computed tomography angiography of the abdomen revealed FMD of the mid SMA with no evidence of FMD in either renal artery.
14 July 2022	LVEF was 10%–15%, with apical akinesis, Grade III diastolic dysfunction, and elevated left-sided filling pressures. A moderate-sized left ventricular thrombus was visualized in the apex. CTA of the chest, abdomen, and pelvis revealed a dissection flap within the SMA.
10 November 2022	The patient underwent advanced heart failure therapy evaluation for ischaemic cardiomyopathy with an LVEF of 25%–30%, limited GDMT due to hypotension, and NYHA Class III symptoms. Medical therapy included bisoprolol 5 mg, lisinopril 5 mg, and spironolactone 25 mg; empagliflozin 10 mg was subsequently added, and a single-chamber ICD was implanted after 3 months.
21 March 2023	Cardiopulmonary exercise testing for 10.15 min, peak VO₂ of 16.8 ml/kg/min, RER of 1.03, VE/VCO₂ slope of 45, and VO₂/HR of 10. Genetic testing revealed a heterozygous FLNC variant of uncertain significance.
August 2023	CTA of the abdomen and pelvis showed a short-segment dissection of the proximal SMA, managed conservatively. LVEF was 30%–35% with a persistent apical thrombus. Further ischaemic workup without viable myocardium and no ischaemia. The patient was referred to cardiopulmonary rehabilitation but had no symptomatic improvement with worsening NYHA Class III symptoms.
16 October 2023	Admitted following RHC with RA pressure 20 mmHg, PAP 58/29 mmHg (mean 42), PCWP 13 mmHg, and mixed venous saturation 45%. Fick CO/CI was 3.12 L/min and 1.79 L/min/m². Milrinone was initiated at 0.35 μg/kg/min. Due to further decompensation, an intra-aortic balloon pump (IABP) was placed.
26 October 2023	The patient was listed for heart transplant as Status 2 while on inotropes and mechanical circulatory support with IABP. Considered a poor LVAD candidate due to severe RV failure. HIT antibody was positive; Platelet factor 4 ELISA was negative. Blood type O positive. PRA Class I 25%, Class II 64%, repeat PRA after a week at 3%.
17 November 2023	Post-orthotopic heart transplant, the patient was weaned off dopamine, epinephrine, isoproterenol, and inhaled nitric oxide (20 ppm) within a few days. Transplant serologies showed EBV mismatch (donor positive, recipient negative), CMV positive in both, and toxoplasmosis negative in both. Repeat CMV PCR post-operatively was negative. Immunosuppression included tacrolimus, mycophenolate, and steroids, with induction therapy using intraoperative basiliximab and a second dose on post-operative day. Prophylaxis included valacyclovir for CMV and trimethoprim-sulfamethoxazole.
November 2023	RHC showed RA of 18 mmHg, PAP of 45/21 mmHg (mean 30), PCWP of 22 mmHg, and mixed venous saturation of 63%. Fick CO was 5.6 L/min; CI was 3.3 L/min/m². Endomyocardial biopsy revealed 1R rejection. The hospital course was complicated by pneumonia and pleural effusion, treated with vancomycin and cefepime. Additional complications included left vocal cord paralysis, managed with hyaluronic acid injection, and right hemidiaphragm weakness, managed conservatively.
8 December 2023	The patient was discharged to a local rehabilitation centre on supplemental oxygen. TTE showed normal biventricular function, and RHC demonstrated stable hemodynamics. Explanted heart pathology revealed transmural healed infarction, LV dilation with hypertrabeculation, endocardial thickening, minimal coronary artery disease, and evidence of a prior dissection in the LM coronary artery.

STEMI, ST-elevation myocardial infarction; tPA, thrombolysis with tissue plasminogen activator; PCI, percutaneous coronary intervention; SCAD, spontaneous coronary artery dissection; LM, left main; LAD, left anterior descending; LCX, left circumflex artery; CABG, coronary artery bypass grafting; PET, positron emission tomography; LVEF, left ventricular ejection fraction; TTE, transthoracic echocardiography; CTA, computed tomography angiography; SMA, superior mesenteric artery; FMD, fibromuscular dysplasia; GDMT, guideline-directed medical therapy; NYHA, New York Heart Association; RHC, right heart catheterization; RA, right atrium; PAP, pulmonary artery pressure; PCWP, pulmonary capillary wedge pressure; RV, right ventricle; LV, left ventricle; IABP, intra-aortic balloon pump; HIT, heparin-induced thrombocytopenia; PRA, panel reactive antibody; EBV, Epstein–Barr virus; CMV, cytomegalovirus; CO, cardiac output; CI, cardiac index.

We present a case involving a 43-year-old woman with fibromuscular dysplasia (FMD) who was admitted with a STEMI at a facility without percutaneous coronary intervention (PCI) capabilities. She was treated with thrombolytics and later diagnosed with SCAD, ultimately experiencing cardiogenic shock that required coronary artery bypass grafting (CABG). Unfortunately, she subsequently developed progressive heart failure, leading to multiple hospitalizations and ultimately requiring heart transplantation 2 years later. This case highlights the diagnostic and therapeutic challenges of managing STEMI in patients with SCAD and underscores the need for early recognition, individualized treatment, and clearer clinical guidance.

## Case summary

**Figure 1:**
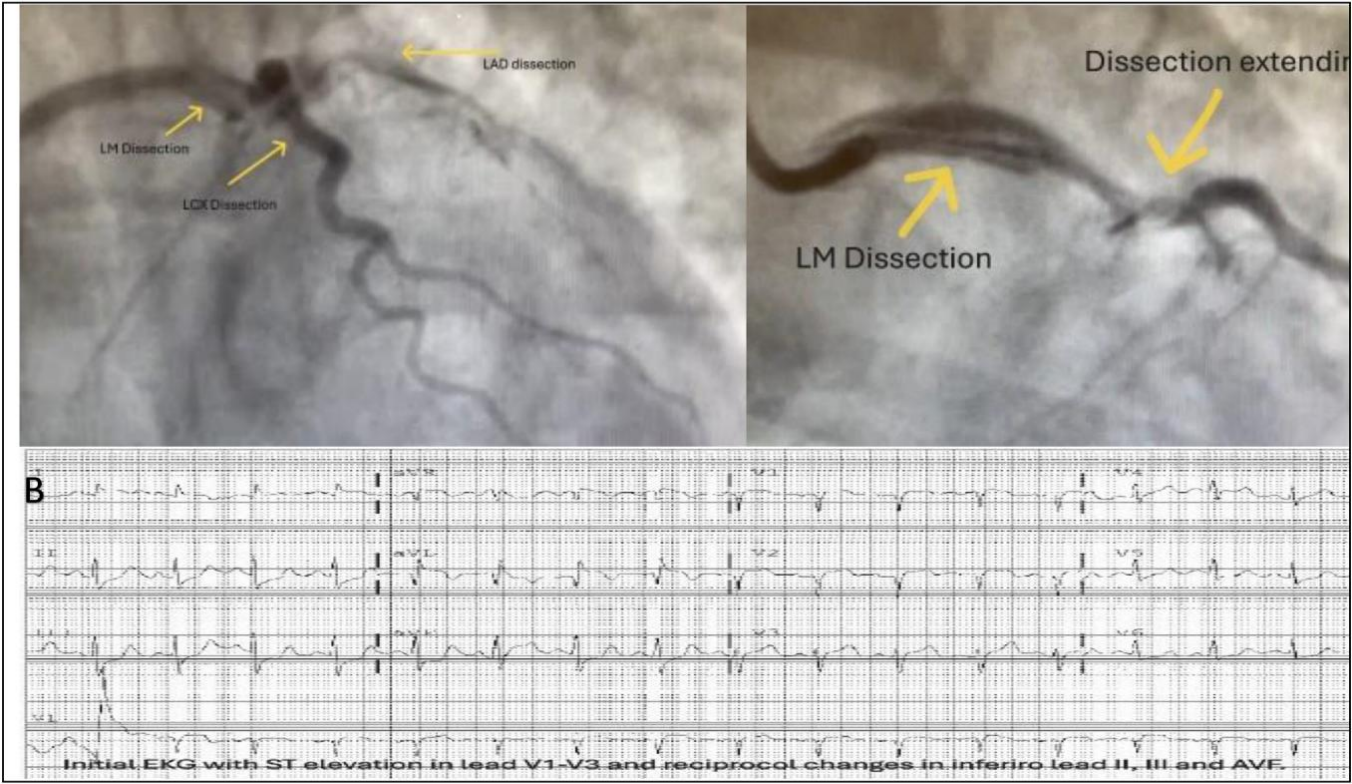
Coronary angiogram showing coronary artery dissection of the left main, left anterior descending, and left circumflex arteries. B—initial electrocardiogram (EKG) with ST elevation in lead V1–V3 and reciprocal changes in inferior leads II, III, and AVF.

A 43-year-old woman with hypertension, asthma, hypothyroidism, depression, and a family history of aortic aneurysm presented with chest pain and was diagnosed with anterolateral ST-elevation myocardial infarction (STEMI), (*Figure 1 EKG*) complicated by respiratory failure and encephalopathy. The patient received a tissue plasminogen activator (tPA) and started on heparin. She developed respiratory failure requiring intubation and remained on mechanical ventilation. The patient was then transferred to a percutaneous coronary intervention (PCI) centre. Radial approach left heart catheterization (LHC) showed spontaneous coronary artery dissection (SCAD) of the left main (LM), left anterior descending (LAD), and left circumflex (LCx) arteries (*Figure 1*). The patient subsequently developed cardiogenic shock requiring temporary mechanical circulatory support. Impella CP was placed via the right femoral artery with a 3.5 L cardiac output, and the left ventricle end-diastolic pressure (LVEDP) was 31. She underwent an emergent three-vessel bypass vein graft to LAD, obtuse marginal (OM), and a large diagonal branch. During surgery, the dissection was confirmed involving the LM, LAD, and LCX (*Figure 1*). Left internal mammary artery (LIMA) dissection was confirmed intraoperatively. She remained on pressors and inotropes. She underwent reoperation the next day with bleeding and required multiple blood product transfusions. She was weaned from Impella and mechanical ventilation in a few days, encephalopathy improved, and the brain MRI was unremarkable. She was also diagnosed with left ventricular (LV) thrombus post-operatively and remained on long-term anticoagulation. She had a prolonged cardiac intensive care unit (ICU) course and ultimately was discharged to acute rehab followed by cardiopulmonary rehabilitation.

**Figure 2:**
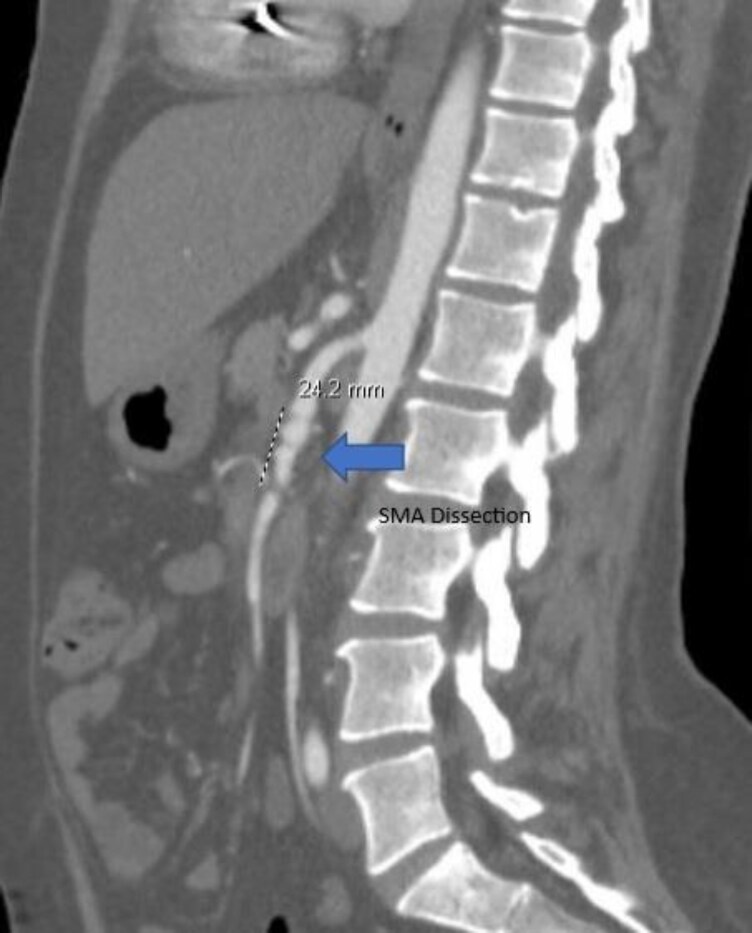
Segment of superior mesenteric artery dissection in a patient with spontaneous coronary artery disease.

**Figure 3:**
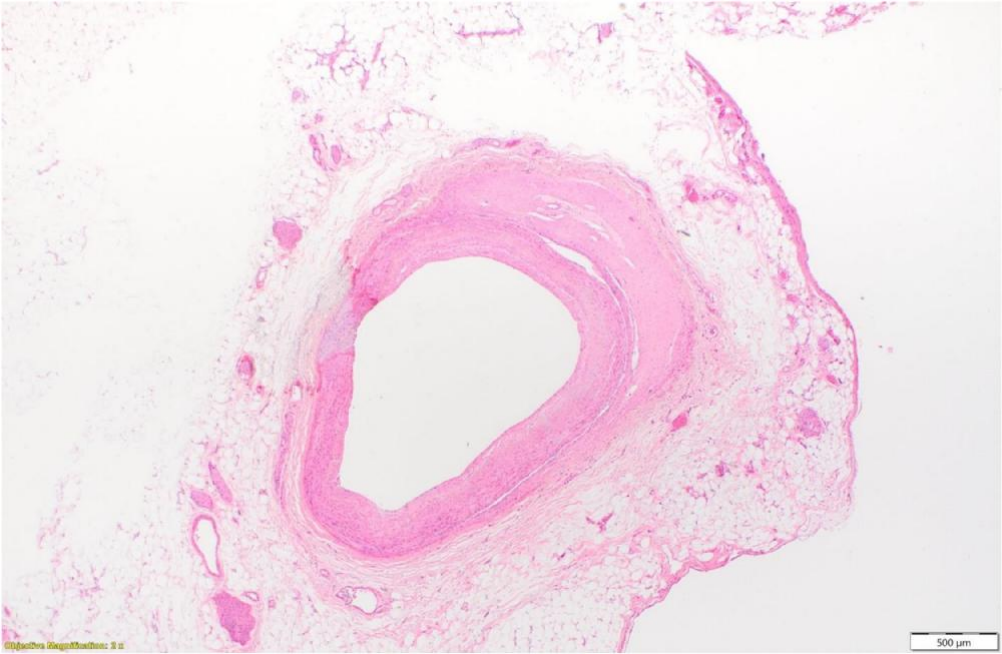
The pathology of the explanted heart showed left ventricular dilatation, prominent endocardial thickening with hypertrabeculation, an area of dissection in the left main coronary artery, and a transmural healed infarction involving the anterior wall, septum, and part of the lateral wall.

Even after 15 months, the patient continued to have poor functional status due to heart failure symptoms, was unable to optimize goal-directed medical therapy (GDMT) with low blood pressure, and was referred for a heart transplant at our centre. CTA chest, abdomen, and pelvis were noted with dissection of the superior mesenteric artery (SMA) (*Figure 2*) and had ANA+ consistent with fibromuscular dysplasia. Vascular surgery evaluated for SMA dissection and managed conservatively. Her genetic testing was heterozygous for the FLNC allele reported as uncertain significance. Transthoracic echocardiography showed LV ejection fraction (LVEF) of 20%–25% with ischemic workup reveiling no reversible perfusion defects and NYHA Class III symptoms. Right heart catheterization showed elevated right-sided filling pressures and low cardiac index (1.7 L/min/m^2^). The patient was admitted in cardiogenic shock on inotrope unable to tolerate higher doses and further supported with an intra-aortic balloon pump and listed for heart transplant UNOS Status 2. She was successfully transplanted after 2 weeks. Post-transplant course required reintubation with hypercapnia, subsequent noted for vocal cord paralysis requiring filling injection, weak right diaphragm, and pneumonia treatment. The explanted heart pathology was reported as minimal coronary artery disease with an area of old dissection in the left main coronary artery section (*Figure 3*). All of the sequences of events are provided in the summary figure.

## Discussion

### Epidemiology and pathophysiology

Spontaneous coronary artery dissection (SCAD) is increasingly recognized as a cause of myocardial infarction (MI) in women under 50, accounting for up to one-third of cases in this age group. It is the leading cause of pregnancy-associated MI, responsible for approximately 15% of such cases and often associated with more complications. However, SCAD represents <1% of all acute MIs in the general population, including men.^1^ Most SCAD patients are women aged 47–53, typically with fewer traditional risk factors than those with atherosclerotic MI. In the Canadian SCAD Cohort Study, 88.5% of the 750 enrolled patients were women, with a mean age of 51.7 years. Over half were post-menopausal, and only 4.5% had peripartum SCAD—highlighting that SCAD is not limited to reproductive age and can occur outside of pregnancy.^2^

The pathophysiology of SCAD involves spontaneous separation within the coronary arterial wall, leading to an intramural haematoma or intimal tear, which compromises blood flow and causes ischaemia. This multifactorial disease is influenced by arteriopathies like FMD, hormonal factors, genetic predisposition, and emotional or physical stressors. FMD is present in 25%–86% of SCAD patients, primarily affecting women.^1^ While rare genetic disorders account for ∼5%–8% of cases, most SCAD instances are sporadic. Recent studies have identified 16 loci associated with SCAD, highlighting the role of genes involved in vascular structure. Hormonal influences, especially during pregnancy, remain poorly understood, while emotional stress triggers nearly half of SCAD cases and physical stressors, such as intense exercise, contribute to about one-third. Male patients often report physical triggers and less frequently have FMD or psychiatric comorbidities compared to females.^1,3,4^

### Diagnostic pitfalls

SCAD should be suspected in young or middle-aged women presenting with ACS without traditional cardiovascular risk factors like smoking, diabetes, hypertension, hyperlipidaemia, family history of premature CAD, or sudden death. Clinical history often includes recent emotional or physical stressors and associated conditions such as fibromuscular dysplasia or recent pregnancy. Electrocardiographic findings in SCAD are non-specific and may mimic other ACS presentations, including ST-segment elevation or depression and T-wave inversions. Troponin levels may initially be normal in a subset of patients, underscoring the need for clinical vigilance. Coronary angiography remains the primary diagnostic modality. Features suggestive of SCAD include long, smooth, non-atherosclerotic stenosis, radiolucent lumens, or signs of intramural haematoma. Three angiographic patterns are described: classic dissection with multiple lumens, diffuse narrowing from haematoma, and focal stenoses mimicking atherosclerosis with a fourth type for total occlusion. When angiographic findings are inconclusive, intracoronary imaging, intravascular ultrasound (IVUS), and optical coherence tomography (OCT) may assist, though they carry procedural risks.^1,5^

### Thrombolysis in spontaneous coronary artery dissection: evidence and controversies

Thrombolysis is discouraged in SCAD as the underlying vessel pathology is typically an intramural haematoma or intimal tear and is particularly susceptible to harm from fibrinolytic therapy. This increases the risk of dissection extension, worsening ischaemia, cardiac tamponade, and the need for emergency surgery.^6^ While meta-analyses from observational data show no mortality benefit, they suggest a higher rate of complications with thrombolysis in SCAD.^7,8^ Current guidelines advise against thrombolysis in suspected SCAD.^4,9^ However, in non-PCI-capable settings, it may be cautiously considered in cases of diagnostic uncertainty where atherothrombotic STEMI is strongly suspected, ideally with immediate plans for transfer to a PCI centre. In such scenarios, early recognition and individualized clinical judgment are essential to avoid misdiagnosis and potential harm.

### Revascularization in spontaneous coronary artery dissection

Most cases of SCAD are managed conservatively due to the potential for spontaneous vessel healing and the increased complication rates associated with invasive strategies. Percutaneous coronary intervention (PCI) poses technical challenges and is often associated with low success rates due to the fragility of the arterial wall and the risk of dissection propagation.^1^ CABG is rarely indicated and is typically reserved for patients with haemodynamic instability, ongoing ischaemia, left main or proximal multivessel dissections as in our case, or failed PCI.^6,10^ The incidence of cardiogenic shock in SCAD ranges from 1.2% to 15.9% and is associated with a higher risk of use of MCS, major bleeding, blood transfusion, and respiratory failure.^11^ However, the long-term efficacy of CABG may be limited due to spontaneous healing of native arteries, which can lead to competitive flow and subsequent graft failure.^4,10^ Observational studies have shown no consistent mortality benefit for revascularization over conservative therapy.^12^ Severe or complex presentations such as those progressing to cardiogenic shock must be individualized and guided by clinical judgment.

### Long-term outcomes and the role of fibromuscular dysplasia in spontaneous coronary artery dissection

While SCAD often resolves with conservative management, a subset of patients experience a progressive course marked by recurrent ischaemic events and heart failure and rarely require heart transplantation. This case reflects such a trajectory of multivessel SCAD deterioration despite CABG. FMD, present in up to 86% of SCAD patients, is associated with systemic arteriopathy and increased long-term risk, prompting guideline-recommended vascular imaging from head to pelvis. Coronary CT angiography aids in surveillance for recurrent or residual lesions. The detection of an FLNC variant, typically linked to dilated cardiomyopathy, suggests overlapping myocardial and vascular pathology. Genetic evaluation is advised for patients with progressive disease or suggestive family history. These findings underscore the need for personalized follow-up strategies and further research into the interplay of FMD, genetic predisposition, and SCAD progression.

## Conclusion

Our case is unique due to its prolonged and complex clinical course, beginning with the administration of tPA with the initial presentation of an acute STEMI. Subsequent investigations revealed SCAD that culminated in cardiogenic shock necessitating emergent coronary artery bypass grafting (CABG). Most reported SCAD cases are resolved with conservative management; some may require revascularization or mechanical circulatory support, either for recovery or as a bridge to transplantation during the same admission. However, our patient experienced a protracted 2-year trajectory marked by progressive heart failure, ultimately leading to an orthotopic heart transplant. This extended course underscores the potential for SCAD to result in chronic myocardial dysfunction, a progression not commonly observed in the literature. This case highlights the critical importance of early and accurate diagnosis of SCAD, particularly in young women presenting with acute coronary syndrome. It also emphasizes the need for awareness regarding the potential complications of thrombolytic therapy in these situations. Furthermore, ongoing research is essential to establish a connection between FMD, genetics, and SCAD, which will help in understanding the underlying mechanisms and long-term outcomes.

## Data Availability

The data underlying this article are available in the article and in its online supplementary material.

## Lead author biography

Dr. Bishow Paudel completed medical school at Manipal College of Medical Sciences in Pokhara, Nepal. He has several years of clinical experience in internal medicine and cardiology after residency at the University of Maryland Midtown Campus. He recently completed an advanced heart failure and transplant cardiology fellowship at the University of Alabama at Birmingham and is currently an advanced cardiovascular imaging fellow at the University of Virginia (UVA) Health.

## Notes

**Consent:** The authors confirm that written consent for the submission and publication of this case report, including images and associated text, has been obtained from the patient by COPE guidance.

**Funding:** B.P. has current advanced cardiovascular imaging training at the University of Virginia supported by NIH T32 Program—T32EB003841.

## References

[1] Hayes SN, Kim CESH, Saw J, Adlam D, Arslanian-Engoren C, Economy KE, et al Spontaneous coronary artery dissection: current state of the science: a scientific statement from the American Heart Association. Circulation 2018;137:e523–e557. 29472380 10.1161/CIR.0000000000000564 PMC5957087

[2] Saw J, Starovoytov A, Aymong E, Inohara T, Alfadhel M, McAlister C, et al Canadian spontaneous coronary artery dissection cohort study: 3-year outcomes. J Am Coll Cardiol 2022;80:1585–1597. 36265953 10.1016/j.jacc.2022.08.759

[3] Kim SK, Wing-Lun E, Chandrasekhar J, Puri A, Burgess S, Ford TJ, et al The Australian New Zealand Spontaneous Coronary Artery Dissection (ANZ-SCAD) registry—a multi-centre cohort study: protocol, background and significance. Heart Lung Circ 2022;31:1612–1618. 36180304 10.1016/j.hlc.2022.08.018

[4] Adlam D, Alfonso F, Maas A, Vrints C; Writing Committee. European Society of Cardiology, acute cardiovascular care association, SCAD study group: a position paper on spontaneous coronary artery dissection. Eur Heart J 2018;39:3353–3368. 29481627 10.1093/eurheartj/ehy080 PMC6148526

[5] Adlam D, Tweet MS, Gulati R, Kotecha D, Rao P, Moss AJ, et al Spontaneous coronary artery dissection: pitfalls of angiographic diagnosis and an approach to ambiguous cases. JACC Cardiovasc Interv 2021;14:1743–1756. 34412792 10.1016/j.jcin.2021.06.027 PMC8383825

[6] Tweet MS, Hayes SN, Pitta SR, Simari RD, Lerman A, Lennon RJ, et al Clinical features, management, and prognosis of spontaneous coronary artery dissection. Circulation 2012;126:579–588. 22800851 10.1161/CIRCULATIONAHA.112.105718

[7] Martins JL, Afreixo V, Santos L, Costa M, Santos J, Gonçalves L. Medical treatment or revascularisation as the best approach for spontaneous coronary artery dissection: a systematic review and meta-analysis. Eur Heart J Acute Cardiovasc Care 2018;7:614–623. 28452228 10.1177/2048872617706502

[8] Kim ESH . Spontaneous coronary-artery dissection. N Engl J Med 2020;383:2358–2370. 33296561 10.1056/NEJMra2001524

[9] Rao SV, O’Donoghue ML, Ruel M, Rab T, Tamis-Holland JE, Alexander JH, et al 2025 ACC/AHA/ACEP/NAEMSP/SCAI guideline for the management of patients with acute coronary syndromes: a report of the American College of Cardiology/American Heart Association joint committee on clinical practice guidelines. Circulation 2025;151:e771–e862. 40014670 10.1161/CIR.0000000000001309

[10] Lei K, Yee B, Dicaro MV, Mubder M, Altaweel O, Choudhury AH. Spontaneous coronary artery dissection in a patient with cardiogenic shock: to revascularize or not to revascularize? Cureus 2024;16:e55050. 38550440 10.7759/cureus.55050 PMC10977166

[11] Krittanawong C, Qadeer YK, Ang SP, Wang Z, Alam M, Sharma S, et al Clinical outcomes of cardiogenic shock due to spontaneous coronary artery dissection versus cardiogenic shock due to coronary artery disease. Crit Pathw Cardiol 2024;23:141–148. 38467033 10.1097/HPC.0000000000000354

[12] Yang C, Offen S, Saw J. What is new in spontaneous coronary artery dissection? CJC Open 2024;6:417–424. 38487071 10.1016/j.cjco.2023.10.007 PMC10935686

